# Development and Validation of the Promising PPAR Signaling Pathway-Based Prognostic Prediction Model in Uterine Cervical Cancer

**DOI:** 10.1155/2023/4962460

**Published:** 2023-05-31

**Authors:** Yan Zhang, Xing Li, Jun Zhang, Lin Mao, Zou Wen, Mingliang Cao, Xuefeng Mu

**Affiliations:** Department of Obstetrics and Gynecology, Renmin Hospital of Wuhan University, Wuhan, China

## Abstract

A ligand-activated transcription factor, peroxisome proliferator-activated receptor (PPAR) regulates fatty acid uptake and transport. In several studies, upregulation of PPAR expression/activity by cancer cells has been associated with cancer progression. Worldwide, cancer of the cervix ranks fourth among women's cancers. Angiogenesis inhibitors have improved treatment for recurrent and advanced cervical cancer since their introduction 5 years ago. In spite of that, the median overall survival rate for advanced cervical cancer is 16.8 months, indicating that treatment effectiveness is still lacking. Thus, it is imperative that new therapeutic methods be developed. In this work, we first downloaded the PPAR signaling pathway-related genes from the previous study. In addition, the single-sample gene set enrichment analysis (ssGSEA) algorithm was applied to calculate the PPAR score of patients with cervical cancer. Furthermore, cervical cancer patients with different PPAR scores show different sensitivity to immune checkpoint therapy. In order to screen the genes to serve as the best biomarker for cervical cancer patients, we then construct the PPAR-based prognostic prediction model. The results revealed that PCK1, MT1A, AL096855.1, AC096711.2, FAR2P2, and AC099568.2 not only play a key role in the PPAR signaling pathway but also show good predictive value in cervical cancer patients. The gene set variation analysis (GSVA) enrichment analysis also proved that the PPAR signaling pathway is one of the most enriched pathways in the prognostic prediction model. Finally, further analysis revealed that AC099568.2 may be the most promising biomarker for the diagnosis, treatment, and prognosis in cervical cancer patients. Both the survival analysis and Receiver Operating Characteristic curve demonstrated that AC099568.2 plays a key role in cervical cancer patients. However, to our knowledge, this is the first time a study focused on the role of AC099568.2 in cervical cancer patients. Our work successfully revealed a new biomarker for cervical cancer patients, which also provides a new direction for future research.

## 1. Introduction

Each year, approximately 500,000 women are diagnosed with invasive uterine cervical cancer (UCC) worldwide, resulting in 273,000 deaths [1]. It is estimated that over 70% of cancer patients have reached a very advanced stage of their illness [2]. It is reported that 604,127 women worldwide will be diagnosed with cervical cancer by 2020 [3]. There could be approximately 7 million fewer cases of human papillomavirus (HPV) over the next half-century with screening campaigns and broad-spectrum vaccinations for HPV [4]. According to recent guidelines released by the International Federation of Gynecological Ecology and Obstetrics (FIGO), a variety of imaging tools, surgery, and pathology can be used to stage cervical cancer [5]. Given the high costs of additional tests, a clinical approach is still considered acceptable in low- and middle-income countries [6]. Although HPV infection is ubiquitous and a major etiological factor in the carcinogenesis process, it is not always detectable in all patients with UCCs [7]. Approximately 75% of cervical cancer patients develop polypoidal exophytic masses inside the ectocervix caused by squamous cell carcinoma [8]. There are also instances where the endocervix may become dilated due to ulcerations, barrel adenocarcinomas, or adenosquamous cell carcinomas, which originate from the columnar epithelium [9]. There are several routes of spread, including the direct extension to the vaginal mucosa, the adjacent parametrial tissues, the bladder, or the rectum [10]. A growing body of knowledge is available about the oncology, tumor biology, and tumor morphology of cervical cancer at present. This field is also currently interested in identifying genetic, molecular, and immunohistochemical markers as early detection tools for precancerous lesions and neoplastic processes. As part of oncology, a biomarker is often a gene, DNA, RNA, protein, enzyme, antigen, or other cellular and biological product [11]. There is evidence that these lesions may occur at various stages of carcinogenesis under the influence of therapy. Many modern reviews and articles have discussed these lesions [12].

Since 1990, Issemann and Green have been discovering ligand-activated transcription factors called peroxisome proliferator-activated receptors (PPARs) [13]. There are three different subtypes of PPAR, PPAR *α*, and PPAR *β*/*δ*, which are located on different chromosomes and encoded by specific genes [14]. In spite of their significant homology, these three proteins differ in their tissue distribution, an affinity for ligands, and biological function [15]. Many modern reviews and articles on carcinogenesis describe how these lesions can be detected at various stages of carcinogenesis, as well as how therapy can influence their development [16]. However, few studies focused on the correlations between PPAR signaling pathways and UCC. Therefore, we aim to explore the potential association between UCC and PPAR signaling pathways by bioinformatics analysis.

The Cancer Genome Atlas (TCGA) database was used to obtain expression data for this study to investigate the role of PPAR signaling pathways in UCC. In addition, the single-sample gene set enrichment analysis (ssGSEA) algorithm was applied to explore the score of PPAR signaling in patients with UCC. A Gene Ontology (GO) enrichment analysis and a Kyoto Encyclopedia of Genes and Genomes (KEGG) enrichment analysis were also conducted in order to identify potential pathways closely related to the key genes. Finally, we decided to explore the potential biomarkers for better prognosis prediction of patients with UCC.

## 2. Methods

### 2.1. Dataset Downloaded

Data on mRNA expression and clinical information were downloaded from the Cancer Genome Atlas database for UCC patients. In addition, the genes that are closely associated with the PPAR were also obtained from the previous studies.

### 2.2. Tumor Immune Estimation Resource Analysis

The Tumor Immune Estimation Resource (TIMER) software program (https://cistrome.shinyapps.io/Timer/) provides a comprehensive approach to analyze immune infiltration in different cancer types. An analysis of TIMER was performed to determine whether immune cell infiltration was related to the level of expression of the immune-related cells.

### 2.3. Single-Sample Gene Set Enrichment Analysis

For each tumor case, an individual score was calculated using ssGSEA. In ssGSEA, ranking-based GSEA methods are used to compute overexpression measures for genes of interest relative to other genes in the genome. Based on log-transformed data from RNA-Seq or microarray experiments, ssGSEA scores were calculated. In the next step, we classified UCCs according to related pathways (ssGSEA scores) and analyzed both tumor purity and immune scores for each patient.

### 2.4. The Enrichment Pathway Analysis Based on the Key Genes

Using functional enrichment, the data were further analyzed to confirm the potential functions of the potential targets. GO is widely used to annotate genes with their functions, especially molecular functions (MF), biological pathways (BP), and cellular components (CC). Analyzing gene function and related high-level genome function information using KEGG enrichment analysis is practical and useful. An analysis of the GO function of potential mRNAs and enrichment of KEGG pathways was performed using the ClusterProfiler package in R to better understand the oncogenic functions of target genes.

### 2.5. Construction of the Prognostic Prediction Model of the PPAR Signaling Pathways

Module members (MM) represent gene expression profiles that are correlated with genes that belong to the module. We then performed univariate analyses of each gene in the module to identify genes associated with the prognosis that were significantly associated. We used COX regression based on the least absolute shrinkage and selection operator (LASSO) to further narrow down the candidate biomarkers for immunization prognosis using the “glmnet” R package. Using the “survminer” R package, samples were divided into low-risk and high-risk groups based on a bivariate model with nonzero coefficients. R was also used to perform the survival analysis.

### 2.6. Immune Cell Infiltration Analysis

An analysis of RNA-seq data from UCC patients in different subgroups was conducted to determine the relative proportions of 22 immune infiltrating cells. To determine whether immune cell infiltration and gene expression are related, Spearman correlation analysis was conducted.

### 2.7. Gene Set Variation Analysis

Gene set variation analysis (GSVA), an unsupervised, non-parametric method, was used to evaluate gene set enrichment. As a result of scoring the genes of interest, followed by determining the biological function of the sample, changes at the gene level were transformed into changes at the pathway level in this study. In the present study, gene sets were retrieved using the molecular signatures database (version 7.0). The GSVA algorithm was used to evaluate a wide range of samples for potential biological function changes.

### 2.8. Gene Set Enrichment Analysis

Gene sets were retrieved from MSigDB (http://www.gsea-msigdb.org/gsea/downloads.jsp). In order to identify enriched GO terms from the gene sets, GSEA was performed using the 50 best terms selected from each subtype.

## 3. Results

### 3.1. The ssGSEA Algorithm Was Used to Obtain the PPAR Signaling Score for UCC Patients

**Figure 1:**
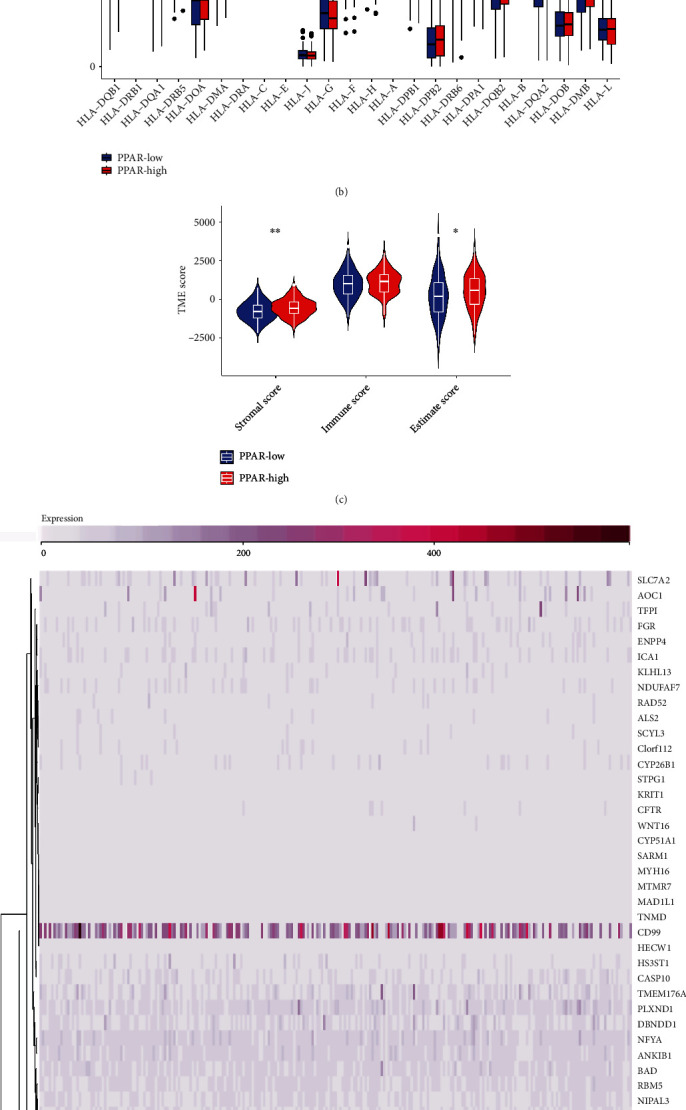
(a) The heat map reveals the results of the ssGSEA algorithm; (b) the different expression levels of HLA-related genes between low- and high-PPAR groups; (c) the different immune-related score between low- and high-PPAR groups; (d) the heat map demonstrated the differentially expressed genes between low- and high-PPAR groups; (e) the volcano map demonstrated the differentially expressed genes between low- and high-PPAR groups.

On the basis of the former study, we successfully obtained the genes that play a key role in the PPAR signaling pathways. Finally, a total of 72 genes were regarded as the genes that are closely associated with the PPAR signaling pathways. Subsequently, by using the ssGSEA algorithm, the patients with UCC were successfully divided into low- and high-PPAR signaling pathways groups. In addition, we also evaluate other pathways, such as cholesterol metabolism, primary bile acid biosynthesis, fat digestion and absorption, glycerolipid metabolism, and regulation of lipolysis in adipocytes. The results demonstrated that the PPAR-high group is associated with the high pathways of cholesterol metabolism, primary bile acid biosynthesis, fat digestion and absorption, glycerolipid metabolism, and regulation of lipolysis in adipocytes (Figure 1(a)). Furthermore, we then explore the correlation between Human Leukocyte Antigen (HLA)-related genes and PPAR score (Figure 1(b)). The results did not show potential associations. In addition, we also discovered that high score of PPAR signaling pathway is associated with a higher stromal score and estimate score (Figure 1(c)). According to the differentially expressed analysis, 290 genes were found to be differentially expressed, including 57 genes that were up-regulated and 233 genes that were down-regulated (Figures 1(d) and 1(e)).

### 3.2. The Potential Pathways That Are Closely Associated with the Differentially Expressed Genes

**Figure 2:**
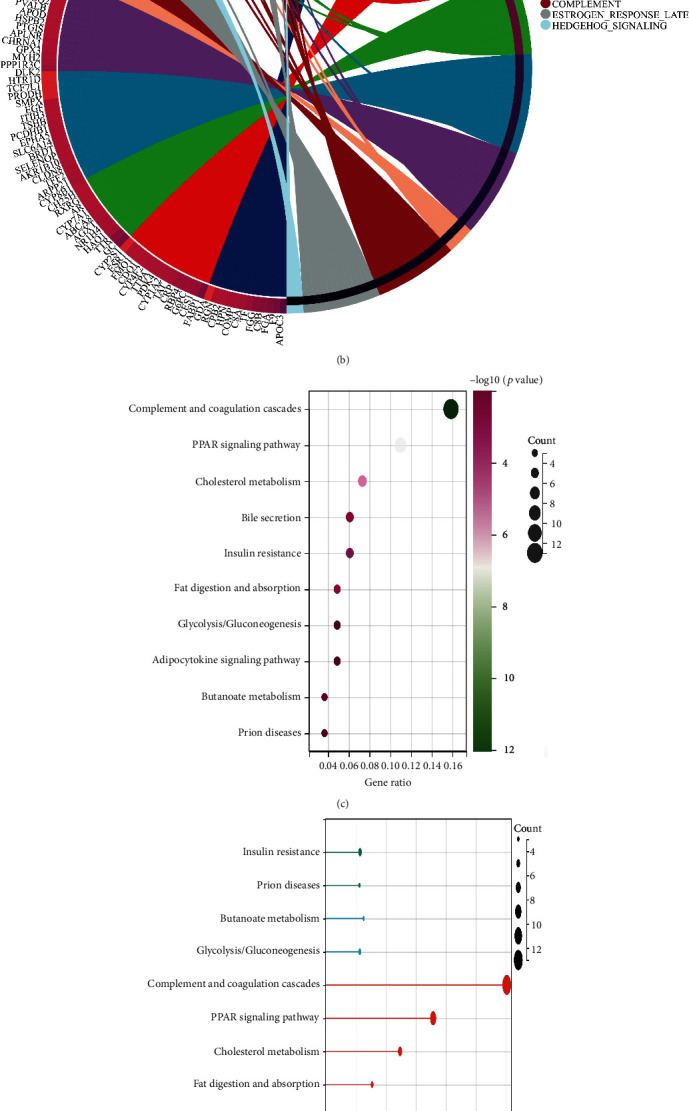
(a, c, and e) The results of KEGG enrichment analysis; (b and d) the enrichment analysis based on the Hallmark terms.

Next, we performed the enrichment pathways analysis based on the 290 different expression genes. The results revealed that complement and coagulation cascades, PPAR signaling pathway, cholesterol metabolism, bile secretion, insulin resistance, fat digestion and absorption, and glycolysis are the most enriched pathways of KEGG terms (Figures 2(a) and 2(b)). Additionally, for Hallmark terms, the most enriched pathways involve coagulation, xenobiotic metabolism, bile acid metabolism, KRAS signaling dn, myogenesis, and angiogenesis (Figures 2(c) and 2(d)).

### 3.3. Evaluation of the Association between PPAR Score and Immune-Related Cells and Immune Checkpoint-Related Genes

**Figure 3:**
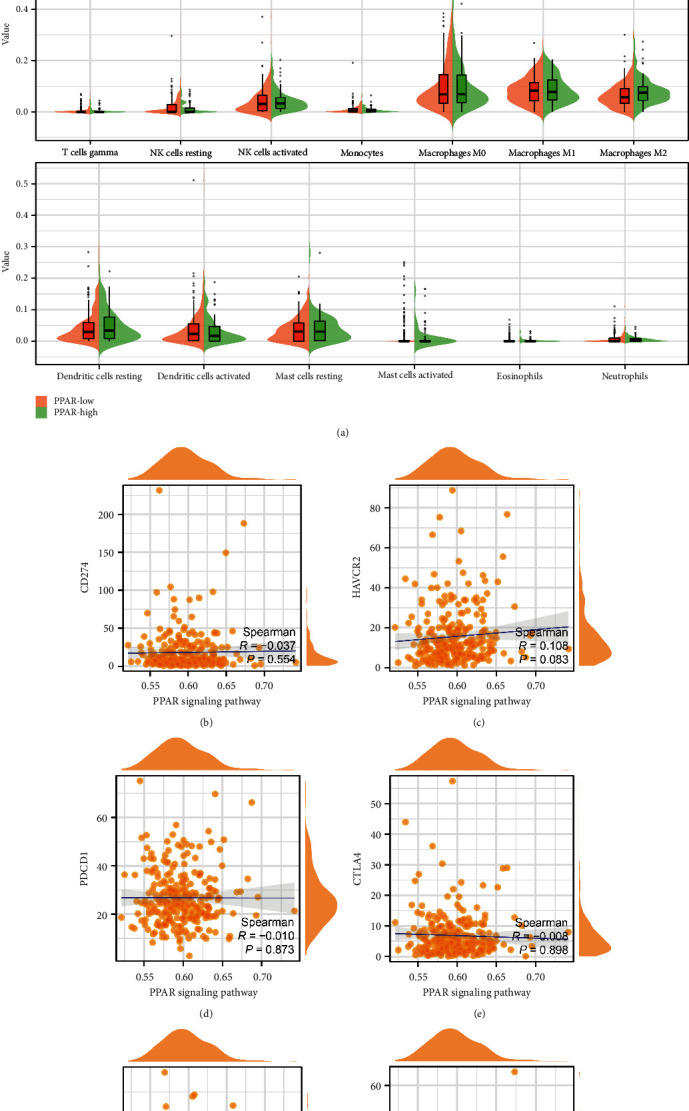
(a) The immune infiltration analysis between low- and high-PPAR groups; the correlation between PPAR score and CD274 (b); HAVCR2 (c); PDCD1 (d); CTLA4 (e); LAG3 (f); PDCD1LG2 (g).

Subsequently, we aim to explore the potential correlation between PPAR score and immune-related cells. A total of 22 types of immune-related cells were identified. The results finally revealed that the lower PPAR score is associated with more infiltration of CD4-activated T cells, while the higher PPAR score is associated with more infiltration of M2 macrophages (Figure 3(a)). In terms of the immune checkpoint genes, the PPAR score is positively associated with HAVCR2, while the PPAR score is negatively associated with CD274, PDCD1, CTLA4, LAG3, and PDCD1LG2 (Figures 3(b), 3(c), 3(d), 3(e), 3(f), and 3(g)).

### 3.4. Construction of the PPAR-Based Prognostic Prediction Model in UCC Patients

**Figure 4:**
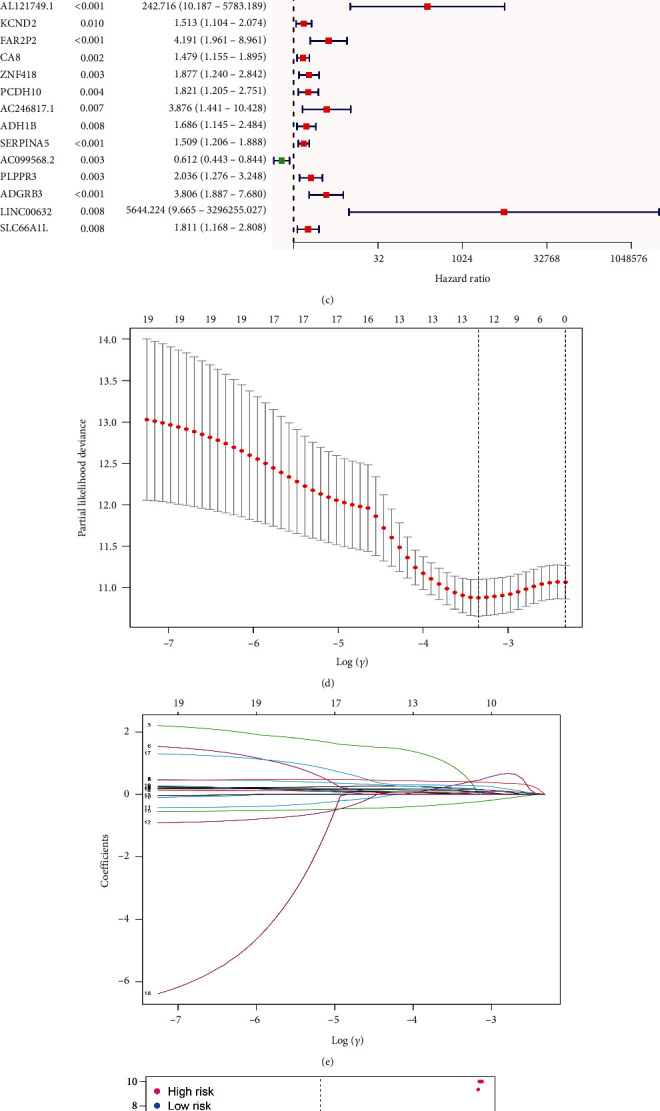
(a and b) The differentially expressed analysis between normal cohort and UCC patients; (c) the results of univariate COX regression analysis; (d and e) the lasso regression analysis; (f) the risk plot between low- and high-risk groups; (g) the survival analysis between low- and high-risk score groups; (h) the time-dependent ROC curve revealed the 1-year, 3-year, and 5-year AUC score of risk score; (i) the calibration score reveals the predictive value of risk score in UCC cohort.

First, we obtained the mRNA expression data, as well as the clinical characteristics of UCC patients. Next, we performed the differentially expressed analysis between UCC patients and normal people. The results demonstrated that a total of 5980 genes showed significant differences, which includes 2033 up-regulated genes and 3947 down-regulated genes (Figure 4(a)). The heat map shows the top 50 differentially expressed genes (Figure 4(b)). Subsequently, we construct the prognostic prediction model based on the overall survival (OS) of UCC patients. The univariate COX regression analysis demonstrated that 19 genes are associated with the prognosis of UCC patients (Figure 4(c)). The LASSO regression analysis and multivariate COX regression analysis were then performed to further explore the biomarkers for the prognosis of UCC patients. The results demonstrated that PCK1, MT1A, AL096855.1, AC096711.2, FAR2P2, and AC099568.2 are mostly associated with the prognosis of UCC patients. We then successfully constructed the PPAR-based prognostic prediction model. Each UCC patient was assigned with the risk score as follows: Risk score = PCK1 × 0.371061037507491 + MT1A × 0.181870631948255 + AL096855.1 × 0.207868336512594 + AC096711.2 × 0.570820588371621 + FAR2P2 × 0.801187844986532 + AC099568.2 × −0.54499718389366 (Figures 4(d) and 4(e)). Based on the risk score, the UCC patients were divided into low- and high-risk groups (Figure 4(f)). The survival analysis revealed that patients with higher risk scores tend to show poorer OS (Figure 4(g)). In addition, the Area Under the Curve (AUC) value of the Receiver Operating Characteristic (ROC) curve was 0.751 at 1 year, 0.731 at 3 years, and 0.675 at 5 years, respectively (Figure 4(h)). The calibration curve proves that PPAR-based prognostic prediction model shows good predictive value in UCC patients (Figure 4(i)).

### 3.5. Validation of the Role of PPAR-Based Prognostic Prediction Model in Immune-Related Cells, Immune Checkpoint Genes, Immune-Related Score, and Clinical Characteristics

**Figure 5:**
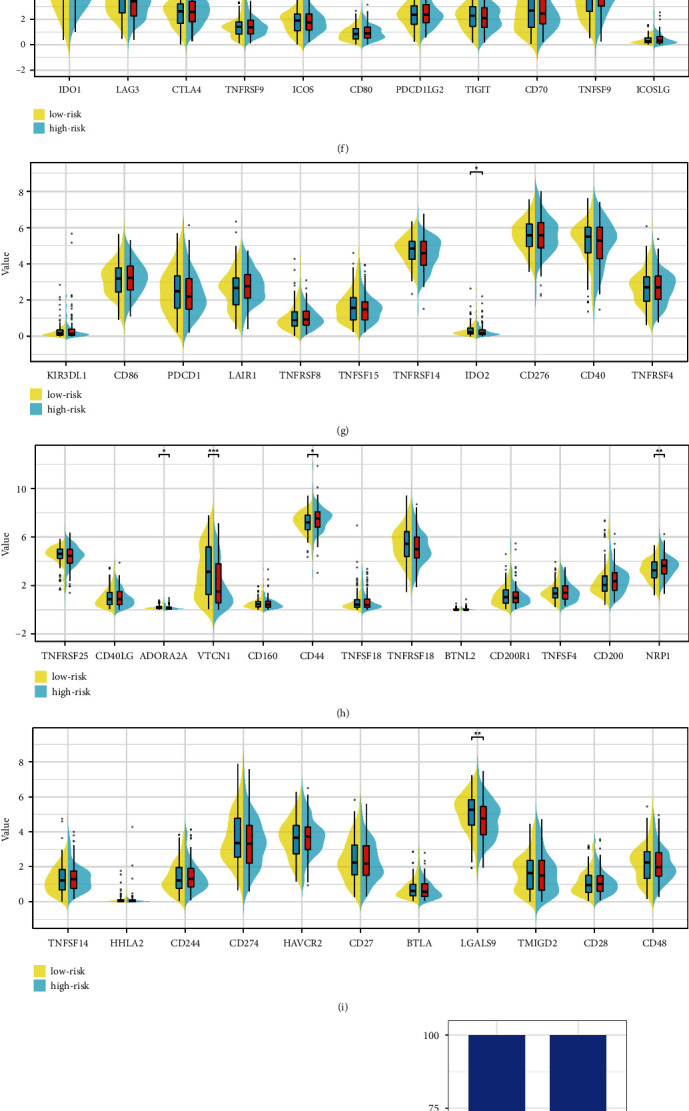
(a) The immune infiltration analysis based on the risk score; the correlation analysis between risk score and cancer-associated fibroblasts (b); endothelial cells (c); macrophages (d); NK cell (e); (f–i) the correlation analysis between risks score and immune checkpoint-related genes; (j) the correlation analysis between risk score and immune-related score; the correlation analysis between risk score and age (k); grade (l); T stage (m); N stage (n).

On the basis of the former analysis, we successfully obtained the PPAR-based prognostic prediction model, which involves six genes (PCK1, MT1A, AL096855.1, AC096711.2, FAR2P2, and AC099568.2). We then performed the immune infiltration analysis. The results demonstrated that the risk score is positively associated with endothelial cells, M2 macrophage, monocyte, Natural Killer (NK) cell, neutrophil, and cancer-associated fibroblasts. However, the risk score is negatively associated with CD8+ naïve T cell, eosinophil, naïve B cell, and T cell follicular helper (Figures 5(a), 5(b), 5(c), 5(d), and 5(e)). The immune checkpoint analysis demonstrated that the risk score is associated with IDO2, ADORA2A, VTCN1, CD44, NRP1, and LGALS9 (Figures 5(f), 5(g), 5(h), and 5(i)). In terms of immune score analysis, the higher risk score is associated with a high stromal score (Figure 5(j)). For clinical characteristics, the UCC patients with the high-risk score are associated with higher age, T stage, and N stage, while the grade is not associated with the risk score (Figures 5(k), 5(l), 5(m), and 5(n)).

### 3.6. Exploration of the Potential Pathways That Are Associated with Risk Score and PPAR-Related Genes

**Figure 6:**
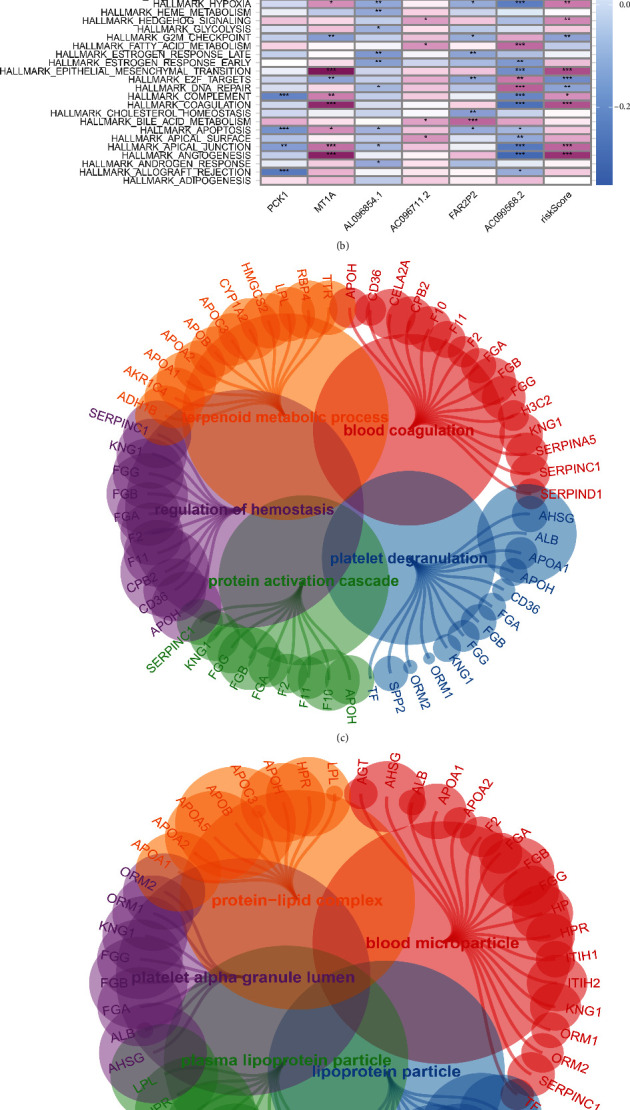
(a) The GSVA analysis based on the KEGG terms; (b) the GSVA analysis based on the Hallmark terms; (c) the GO BP enrichment analysis; (d) the GO CC enrichment analysis; (e) the GO MF enrichment analysis.

Then, we performed the pathway enrichment analysis based on the risk score. The GSVA analysis shows that the calcium signaling pathway, receptor signaling pathway, PPAR signaling pathway, and Transforming Growth Factor (TGF)-beta signaling pathway are the most enriched KEGG terms (Figure 6(a)). For Hallmark terms, angiogenesis, apical junction, coagulation, complement, E2F target, KRAS signaling, and pancreas beta cells are the most enriched pathways. In addition, we also explore the GO enrichment pathways based on the PPAR-related genes (Figure 6(b)). For GO BP analysis, blood coagulation, platelet degranulation, protein activation cascade, regulation of hemostasis, and terpenoid metabolic process are the most enriched pathways (Figure 6(c)). The blood microparticle, lipoprotein particle, plasma lipoprotein particle, protein–lipid complex, and platelet alpha granule lumen are the most enriched GO CC enrichment pathways (Figure 6(d)). In addition, the GO MF enrichment analysis demonstrated that heparin binding, peptidase inhibitor activity, endopeptidase regulator activity, sulfur compound binding, and endopeptidase inhibitor activity are most associated with PPAR-related genes (Figure 6(e)).

### 3.7. AC099568.2 May Play a Key Role in the UCC and PPAR Signaling Pathway

**Figure 7:**
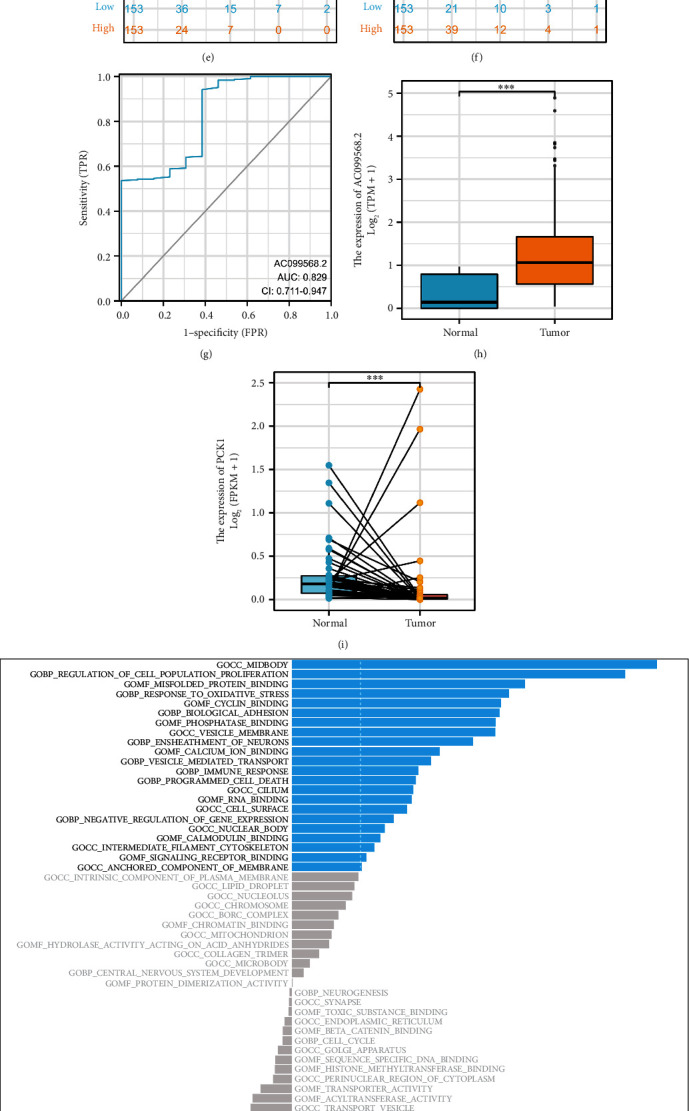
The survival analysis of AC099568.2 (a); FAR2P2 (b); AC096711.2 (c); AL096855.1 (d); MT1A (e); PCK1 (f) in UCC cohort; (g) the ROC curve of AC099568.2 in UCC cohort; (h) the box plot reflects the differentially expressed analysis of AC099568.2; (i) the results of the paired differently expressed analysis; (j) the GSVA analysis of AC099568.2; (k) the GSEA analysis of AC099568.2.

Based on the PPAR-based prognostic prediction model constructed in the previous analysis, we successfully obtained six genes, which may be the biomarkers (PCK1, MT1A, AL096855.1, AC096711.2, FAR2P2, and AC099568.2) for UCC. Subsequently, we performed the survival analysis solely on these six genes. The results demonstrated that the high expression of AC099568.2 is associated with a better prognosis of UCC patients, while the other five genes are not associated with the OS of UCC patients (Figures 7(a), 7(b), 7(c), 7(d), 7(e), and 7(f)). In addition, the ROC curve also proved the good predictive value of AC099568.2 in the UCC cohort (Figure 7(g)). Additionally, the expression level of AC099568.2 in UCC patients is higher than normal cohort (Figures 7(h) and 7(i)). Subsequently, the GSVA analysis demonstrated that midbody, regulation of cell population proliferation, misfolded protein binding, response to oxidative stress, and cyclin binding are positively associated with AC099568.2. However, external encapsulating structure, smoothened signaling pathway, kinase binding, microtubule cytoskeleton, and response to xenobiotic stimulus are correlated with the down-regulation of AC099568.2 (Figure 7(j)). In addition, the GSEA analysis revealed that keratinization, NK activation involved in immune response, negative regulation of interleukin 8 productions, and positive regulation of cytoplasmic translation are closely associated with the AC099568.2 (Figure 7(k)).

## 4. Discussion

Every year, thousands of women die from cervical cancer. Approximately 273,000 women die from cervical cancer each year, despite preventive HPV vaccines and conventional cancer treatments [17]. Malignant cells evade immune surveillance by forming tumors, invading, and metastasizing when their immune systems are perturbed [18]. A deeper understanding of the immune system players that suppress or promote cervical cancer is essential to develop more targeted treatments with fewer side effects [19]. Using natural processes of action to stimulate the immune system to fight cancer cells, immunotherapy has become the most desirable method of targeting cancer [20]. It is possible to treat cervical cancer with a variety of immunotherapy approaches, including monoclonal antibodies, immune checkpoint blockade therapy, adoptive cell transfer therapy, and oncolytic viruses [21]. Recent studies have found that PPARs, which are nuclear hormone receptors, may be used as therapeutic targets for a variety of cancers, including lung cancer [22]. Furthermore, PPARs participate in various cellular functions, such as differentiation, proliferation, survival, apoptosis, and motility [23]. Cancer risk is increased when these cellular processes and metabolic disturbances are dysregulated in tumors [24]. In recent years, with the development of bioinformatics analysis, more and more research started to focus on the advantages of bioinformatics analysis in the treatment, prognosis prediction, and diagnosis of cancer patients [25–31]. In this work, we aim to explore the role of PPAR signaling pathways in UCC patients. By using the ssGSEA algorithm, the UCC cohort was successfully divided into PPAR-low and PPAR-high groups. In addition, the differentially expressed analysis revealed a total of 290 PPAR-related genes. The pathway enrichment analysis also proved that the PPAR signaling pathway is one of the most enriched pathways. Cancer prevention and treatment may be improved using PPAR modulators, including agonists and antagonists. A number of factors contribute to cancer risk, including dyslipidemia, obesity, glucose intolerance, and low-grade inflammation. Therefore, PPAR modulators can be used to treat cancer by promoting proliferation, differentiation, and apoptosis of cancerous cells. They have a significant role to play in preventing various types of cancer, such as cancer of the breast, lung, and pancreas.

**Figure 8:**
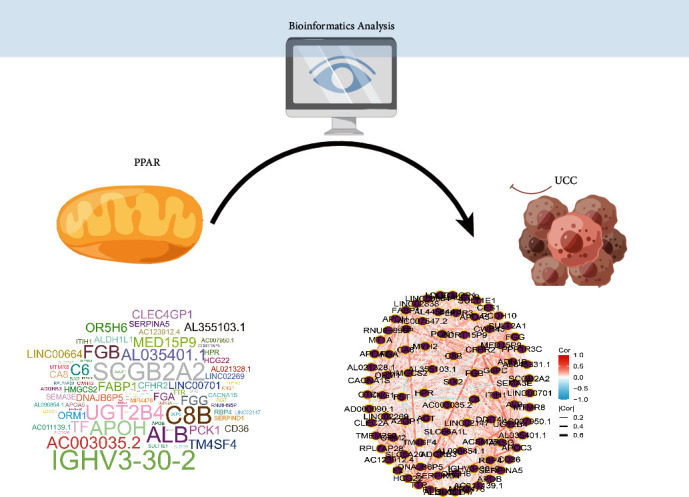
The flow chart of this work.

Subsequently, by constructing the prognostic prediction model based on the PPAR-related genes, we successfully obtained a six-gene-based prognostic prediction model. The survival analysis and ROC curve demonstrated that the PPAR-based model shows good predictive value in UCC patients. In addition, the immune checkpoint analysis demonstrated that the expression level of many immune checkpoint-related genes is closely associated with the PPAR-based risk score, which may indicate that the PPAR signaling pathway map influences immune checkpoint therapy (Figure 8). As shown in the previous study, PPAR*α* acts as a transcription factor influencing intracellular signaling events and cellular metabolism [32]. In conditions of various immunological backgrounds, PPAR-targeted therapies have become more commonly used due to their broad effects on the immune system [33].

Finally, further analysis revealed that AC099568.2 may be the most promising biomarker for the diagnosis, treatment, and prognosis in UCC patients. Both the survival analysis and ROC curve demonstrated that AC099568.2 plays a key role in UCC patients. However, to our knowledge, this is the first time a study focused on the role of AC099568.2 in UCC patients. Our work successfully revealed a new biomarker for UCC patients, which also provides a new direction for future research.

## 5. Conclusion

In this work, we construct the PPAR-based prognostic prediction model. PCK1, MT1A, AL096855.1, AC096711.2, FAR2P2, and AC099568.2 not only play a key role in the PPAR signaling pathway. Further analysis revealed that AC099568.2 may be the most promising biomarker for the diagnosis, treatment, and prognosis in cervical cancer patients.

## Data Availability

The data used to support the findings of this study are included within the article.
